# Personals

**Published:** 1892-08

**Authors:** 


DR. CHAS. D. ROY left Aug. 1st for New York, where he takes a steamer for Liverpool, thence to London. After a short stay in this latter place, he goes direct to Berlin, Vienna and Munich, where for a year or more he will prosecute his studies in the eye, ear and throat. He will also go to Leipsic for a brief stay.

---

DR. R. R. KIME has moved to his new residence, 13 Morrison Ave., where he will shortly be prepared for the reception of a limited number of patients in the line of gynecology and obstetrics, to which he will in future limit his practice. His office will still remain at 63 1-2 Whitehall St.

---

DR. M. ASHBY PURSE, of Savannah, a graduate of the Southern Medical College, and more recently of the College of Physicians and Surgeons, New York, has lately came to Atlanta, where he will locate and engage in the practice of medicine.

Dr. R. O. Cotter, of Macon, has been elected president of the Barnesville Bank, *vice* Hon. R. J. Powell, deceased. Dr. Cotter was a son-in-law of the late Col. Powell. By reason of this new duty, he will move to Barnesville, at least temporarily.

---

Dr. W. E. B. Davis, who for some months past has been associated with Dr. J. B. S. Holmes, of Rome, in a sanitarium for the treatment of diseases of women, has decided to remove to Birmingham, where he originally practiced.

---

The Mississippi Valley Medical Association will hold its eighteenth annual session at Cincinnati Wednesday, Thursday and Friday, October 12th, 13th and 14th. A large attendance and an interesting program are expected.

---

Dr. J. A. Childs, of Atlanta, has been in New York some weeks, pursuing a special course in genito urinary diseases.

---

Dr. K. C. Divine, of Atlanta, has decided that in future he will limit his practice to rectal surgery.

